# Ferroelectric switching in bilayer 3R MoS_2_ via interlayer shear mode driven by nonlinear phononics

**DOI:** 10.1038/s41598-019-50293-y

**Published:** 2019-10-17

**Authors:** Jaehong Park, In Won Yeu, Gyuseung Han, Cheol Seong Hwang, Jung-Hae Choi

**Affiliations:** 1 Center for Electronic Materials, Korea Institute of Science and Technology, Seoul, 02792 Korea; 2 Department of Materials Science and Engineering and Inter-University Semiconductor Research Center, Seoul National University, Seoul, 08826 Korea

**Keywords:** Atomistic models, Nanoscale materials

## Abstract

We theoretically investigate the mechanism of ferroelectric switching via interlayer shear in 3R MoS_2_ using first principles and lattice dynamics calculations. First principle calculations show the prominent anharmonic coupling of the infrared inactive interlayer shear and the infrared active phonons. The nonlinear coupling terms generates an effective anharmonic force which drives the interlayer shear mode and lowers the ferroelectric switching barrier depending on the amplitude and polarization of infrared mode. Lattice dynamics simulations show that the interlayer shear mode can be coherently excited to the switching threshold by a train of infrared pulses polarized along the zigzag axis of MoS_2_. The results of this study indicate the possibility of ultrafast ferroelectricity in stacked two-dimensional materials from the control of stacking sequence.

## Introduction

Ferroelectric two-dimensional (2D) materials are of great importance in realizing non-volatile devices with extreme feature size^1,2^, and possibly with unforeseen functionalities from the unique properties of 2D materials^3,4^. However, studies on the 2D ferroelectrics are yet an emerging field. Only a few materials such as SnTe^5^, α-In_2_Se_3_^6^ have been experimentally shown to work in agreement with theories^7–9^. Transition metal dichalcogenide (TMDC) is another major 2D materials class showing versatile electronic phases ranging from semiconducting or metallic phase to that with topological characteristics^10–12^. Nonetheless, the ferroelectricity of the TMDC is very scarce due to the underlying symmetry of single layer in stable phase, which is either non-polar or centrosymmetric, precluding the electric polarization; i.e. 2H ($P\bar{6}m2$), 1T ($P\bar{3}m1$) and distorted 1T (*P*2/*m*) phases^11^. While theory showed ferroelectric instability of single layer 1T MoS_2_^13^, its realization in the experiment is challenging because MoS_2_ is stable in semiconducting 2H phase rather than metallic 1T structure^14^.

**Figure 1:**
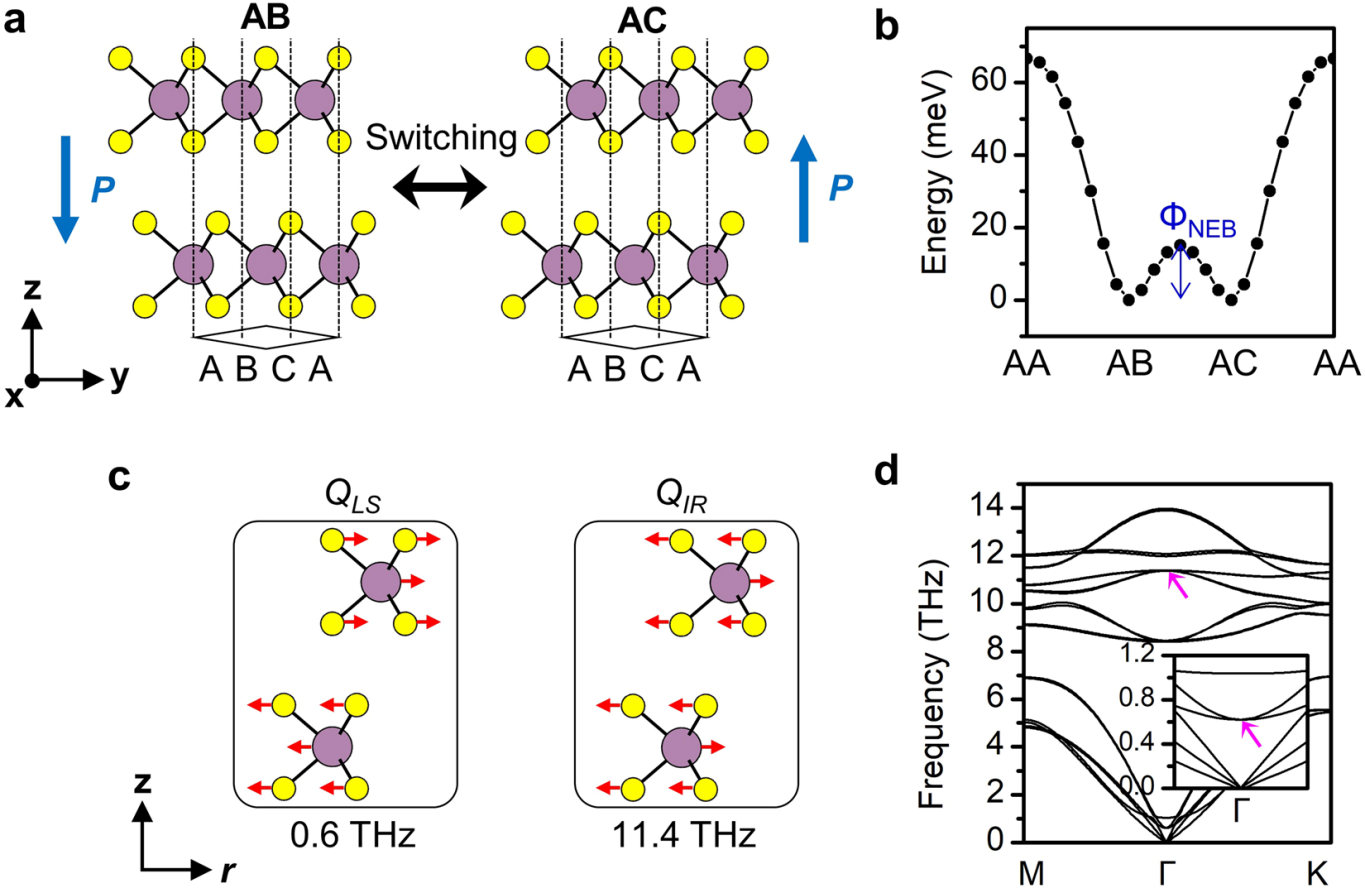
(**a**) Stacking-dependent spontaneous polarization of bilayer 3R MoS_2_. (**b**) Energy of the bilayer structure depending on the stacking sequence. Φ_NEB_ is the ferroelectric switching barrier calculated from the NEB method. (**c**) Displacement patterns of the interlayer shear mode (*Q*_*LS*_) and the infrared mode (*Q*_*IR*_) mode along the in-plane polarization direction *r*. (**d**) Phonon dispersion of the bilayer 3R MoS_2_.The zone-center *Q*_*LS*_ mode at 0.6 THz and *Q*_*IR*_ mode at 11.4 THz are denoted by arrows.

The ferroelectricity is likely to appear in stacked TMDC rather than in single layer form. It was recently shown that the horizontal mirror symmetry of individual layers is broken by the stacking in 3R structure (*P*3*m*1 for finite layers and *R*3*m* for bulk), hence the vertical electric polarization manifests itself in accordance with the global polar symmetry^15^. The direction of polarization depends on the stacking sequence, hence is reversed by the interlayer translation between the AB and AC stackings^15,16^ as shown in Fig. 1a. The possibility of ferroelectric switching in the 3R structure via the interlayer translation has not been explored to date. Meanwhile, the multilayer distorted 1T WTe_2_ showed switching of the polarization^17,18^ and the topological phase^12^ in the recent experiments, probably via the interlayer translation. The stability of the 3R structure MoS_2_ is comparable to that of the 2H structure and can be selectively synthesized among competing polytypes^19^. It is hence a viable candidate for 2D ferroelectrics in which the fascinating phenomena such as high electron mobility^16^ and valleytronics^15,19^ can be explored altogether.

The interlayer translation universally manifests as Raman active low-frequency lattice vibrations in layered 2D crystals due to the weak van der Waals bonds (vdW) between layers^20–22^. Therefore, the ferroelectric switching using an optical field based on ionic Raman scattering is considerably appealing^23–25^. A particular mechanism, called nonlinear phononics, relies on the anharmonic phonon coupling between infrared active and targeted secondary vibrational modes, which displaces the crystal toward the reversal of polarization upon the irradiation of intense terahertz pulse^26,27^. The ionic Raman scattering is distinguished from the conventional Raman scattering which has been used to detect structural characteristics of 2D materials such as stacking structures^28^ and local bonding chemistry^29^. The use of pulse with the mid-infrared frequency within short duration allows the exploration of an extremely intense light field (peak electric field reaching ~600 MVcm^−1^) without the material damage^30–32^. The optical ferroelectric switching is a rapidly growing topic, which will enable the ultrafast and nondestructive way to achieve coherent switching^26,27,33–35^.

In this work, we theoretically show the possibility of the ferroelectric switching of the bilayer 3R MoS_2_ using the intense light pulse through the anharmonic phonon coupling. Density functional theory (DFT) calculations demonstrate that a large amplitude vibration of infrared mode can effectively lower the ferroelectric switching barrier, and induce an unidirectional anharmonic force on the interlayer shear mode along the switching direction. This effect depends on the polarization angle of the incident light pulse with respect to the crystallographic axis of MoS_2_ according to the selection rule. Lattice dynamics simulations indicate the possibility of dynamical ferroelectric switching through the coherent amplification of the interlayer shear mode and the lowering of the energy barrier under the repetitive pulses within a few picoseconds.

## Results

### Electric polarization and switching in bilayer 3R MoS_2_

The bilayer 3R MoS_2_ has the polar point group symmetry of *C*_3v_ with the polar axis along the z-axis. The 3R structure can be constructed by either AB or AC stacking sequence, which develops the spontaneous electric polarization in the opposite direction of each other as shown in Fig. 1a. The magnitude of electric polarization of the bilayer structure was calculated from the Berry phase method as *P* = 0.24 μCcm^−2^, in agreement with previous reports^15,16^. Figure 1b shows the total energy of the bilayer structure (primitive cell consisting of 6 atoms) depending on the stacking sequence calculated from the nudged elastic band (NEB) method. Both the AB and AC stackings are stable and energetically degenerate structures. The AC stacking is obtained from the AB stacking by sliding the upper B layer along the +y direction by 1.82 Å. The interlayer translation over the weak vdW interaction results in the modest energy barrier Φ_NEB_ = 15.0 meV.

The optical switching mechanism was investigated based on the nonlinear phononics^23,24^. The bilayer 3R MoS_2_ has 18 zone-center phonon modes which are decomposed into Γ = 6A_1_ + 6E representations. The singly degenerate A_1_ mode involves out-of-plane motion of atoms, while the doubly degenerate E mode involves in-plane motion of atoms. Figure 1c shows two kinds of E modes relevant to the nonlinear phononics mechanism. The low-frequency mode (Ω_*LS*_ = 0.6 THz in phonon dispersion in Fig. 1d) referred to as the interlayer shear mode (denoted by *Q*_*LS*_) involves the relative motion between adjacent layers along the in-plane polarization axis *r*. Therefore, the *Q*_*LS*_ mode is related to the AB ↔ AC stacking change. The infrared activity of the *v*-th mode is proportional to the square of mode effective charge ${Z}_{v}^{\ast }$^36^. Due to the almost rigid relative ionic motion, the *Q*_*LS*_ mode does not produce net dipole moment as the calculated effective charge ${Z}_{LS}^{\ast }$ = 0.00 *eμ*^−1/2^ (where *e* is the electronic charge and *μ* is the atomic mass unit). The vanishing infrared activity indicates that it is almost impossible to directly excite the *Q*_*LS*_ mode with large amplitude to induce the stacking change. Nonetheless, the anharmonic coupling of *Q*_*LS*_ mode with other infrared active modes can provide an alternative route to control this mode, and the consequent ferroelectric switching. Among the other in-plane modes, only the high-frequency mode (denoted by *Q*_*IR*_) at Ω_*IR*_ = 11.4 THz shows finite effective charge ${Z}_{IR}^{\ast }$ = 0.23 *eμ*^−1/2^, and is the solely infrared active mode under vertical incidence of light.

**Figure 2:**
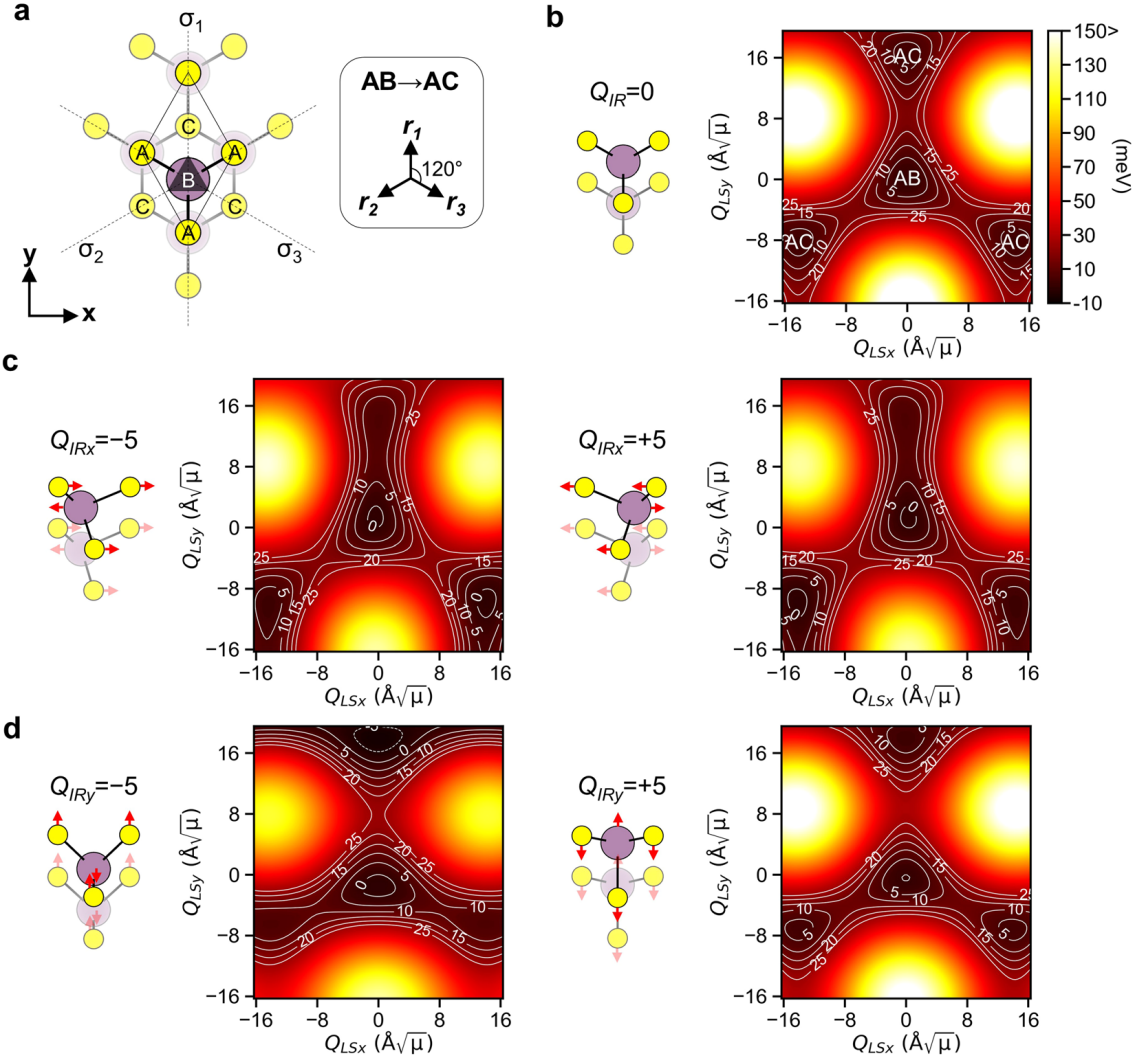
(**a**) Top view of the AB stacked 3R MoS_2_ showing the *C*_3v_ symmetry. In the AB stacking, the S atoms in the upper layer are on the top of the Mo atoms in the lower layer (bottom layer is depicted as dimmed). The x- and y- axis corresponds to the zigzag and armchair axis of MoS_2_, respectively. The AB stacking changes to the AC stacking under the deformation induced by the positive amplitude of *Q*_*LS*_ along the three equivalent directions *r*_1_, *r*_2_ and *r*_3_. (**b**) Potential energy surface *V*(*Q*_*IR*_, *Q*_*LSx*_, *Q*_*LSy*_) on the (*Q*_*LSx*_, *Q*_*LSy*_) coordinates for *Q*_*IR*_ = 0 Å$\sqrt{\mu }$ (*μ* is atomic mass unit). The AB stacking corresponds to the origin (0, 0). Polarization-dependent modulation of the potential energy landscape at (**c**) *Q*_*IRx*_ = ±5 Å$\sqrt{\mu }$ and (**d**) *Q*_*IRy*_ = ±5 Å$\sqrt{\mu }$ via anharmonic coupling.

The normal-mode coordinate *Q*_*v*_ of *v*-th mode is related to the atomic displacement vector ${U}_{i}^{v}=\frac{{Q}_{v}}{\sqrt{{m}_{i}}}{e}_{i}^{v}$, where *m*_*i*_ is the atomic mass of *i*-th atom and ${e}_{i}^{v}$ is the normalized eigenvector of the dynamical matrix. The orthogonal basis sets were chosen to represent the degenerate *Q*_*LS*_ and *Q*_*IR*_ modes as {*Q*_*LSx*_, *Q*_*LSy*_} and {*Q*_*IRx*_, *Q*_*IRy*_}, respectively. They correspond to the linear polarization along the zigzag (x-axis) and armchair (y-axis) axes shown in the top view of the AB stacking of the 3R MoS_2_ in Fig. 2a. The AB stacking deformed by the positive amplitude of *Q*_*LSy*_ = +16.29 Å$\sqrt{\mu }$ (atomic displacement of the each of the adjacent layers in the opposite direction by 0.91 Å) corresponds to the AC stacking. Meanwhile, the deformation by the negative amplitude of the *Q*_*LSy*_ = −16.29 Å$\sqrt{\mu }$ changes the AB stacking into the unstable AA stacking. The positive amplitude of *Q*_*LS*_ along three crystallographically equivalent directions, *r*_1_ (+y direction), *r*_2_ and *r*_3_ directions (−120° and +120° from the +y direction), equally change the AB stacking into AC stacking as shown in Fig. 2a.

The possibility of the ferroelectric switching hinges on how much the *Q*_*LS*_ mode can be amplified along the desired direction for the AB ↔ AC stacking change by the coupling with *Q*_*IR*_. Here, the anharmonic coupling property was investigated from the potential energy surfaces as a function of normal-mode coordinates. The potential energy surfaces *V*(*Q*_*IR*_, *Q*_*LSx*_, *Q*_*LSy*_) for each *Q*_*IRx*_ and *Q*_*IRy*_ were calculated using DFT on 21 × 21 × 23 points with steps of 0.82 Å$\sqrt{\mu }$ for *Q*_*IR*_ and 1.63 Å$\sqrt{\mu }$ for *Q*_*LS*_ modes. The energy surface was then fitted to the polynomial function as

1 $$V({Q}_{IR},{Q}_{LSx},{Q}_{LSy})=\sum _{\{l,m,n\}}{c}_{lmn}{Q}_{IR}^{l}{Q}_{LSx}^{m}{Q}_{LSy}^{n}$$

where *Q*_*IR*_ is either *Q*_*IRx*_ or *Q*_*IRy*_, and *c*_*lmn*_ is the anharmonic coefficient, and ${Q}_{IR}^{l}$, ${Q}_{LSx}^{m}\,\mathrm{and}\,{Q}_{LSy}^{n}$ denote the *l*, *m* and *n* powers of the normal-mode coordinates, respectively. Using this expression, we analyze the effect of the irradiating light pulse with the linear polarization along the x- or y-axis, thus exciting *Q*_*IRx*_ or *Q*_*IRy*_ mode, respectively. Note that the normal-modes in the cartesian basis are classified into the odd parity modes (*Q*_*LSx*_ and *Q*_*IRx*_) and even parity modes (*Q*_*LSy*_ and *Q*_*IRy*_) under the mirror symmetry *σ*_1_ shown in Fig. 2a. The mirror parity imposes the polarization-dependent selection rule on ${Q}_{IRx}^{l}{Q}_{LSx}^{m}{Q}_{LSy}^{n}$ $({Q}_{IRy}^{l}{Q}_{LSx}^{m}{Q}_{LSy}^{n})$ coupling such that *c*_*lmn*_ is nonzero only for *l* + *m* = even (*m* = even). The terms were included up to 15^th^ power terms (*l* + *m* + *n* = 15) in the polynomial function, which fits the DFT potential energy surface accurately. The representative coupling terms are displayed in Table 1.

**Table 1:** The anharmonic coefficient *c*_*lmn*_ for ${Q}_{IR}^{l}{Q}_{LSx}^{m}{Q}_{LSy}^{n}$ coupling terms for each *Q*_*IRx*_ and *Q*_*IRy*_ mode. The values are shown up to 5th power coupling terms in unit of meVÅ^−(*l*+*m*+*n*)^*μ*^−(*l*+*m*+*n*)/2^.

*l*	*m*	*n*	*Q*_*IRx*_	*Q*_*IRy*_	*l*	*m*	*n*	*Q*_*IRx*_	*Q*_*IRy*_
0	0	3	−5.64 × 10^−2^	−5.71 × 10^−2^	1	3	0	1.07 × 10^−3^	
0	0	4	−2.48 × 10^−3^	−2.29 × 10^−3^	1	3	1	1.15 × 10^−4^	
0	0	5	1.22 × 10^−4^	1.22 × 10^−4^	1	4	0		−1.01 × 10^−3^
0	2	0	7.93 × 10^−1^	7.90 × 10^−1^	2	0	0	2.66 × 10^−2^	2.62 × 10^−2^
0	2	1	1.70 × 10^−1^	1.71 × 10^−1^	2	0	1	−6.64 × 10^−2^	1.03 × 10^−1^
0	2	2	−4.92 × 10^−3^	−4.79 × 10^−3^	2	0	2	−3.28 × 10^−3^	−6.17 × 10^−4^
0	2	3	−2.85 × 10^−4^	−2.88 × 10^−4^	2	0	3	5.91 × 10^−4^	−5.58 × 10^−5^
0	4	0	−2.38 × 10^−3^	−2.30 × 10^−3^	2	2	0	3.98 × 10^−4^	−6.14 × 10^−3^
0	4	1	−3.76 × 10^−4^	−4.00 × 10^−4^	2	2	1	1.30 × 10^−4^	−1.13 × 10^−3^
1	0	1		1.81 × 10^−2^	3	0	0		−5.66
1	0	2		5.43 × 10^−2^	3	0	1		−1.5710^−2^
1	0	3		2.36 × 10^−3^	3	0	2		−5.75 × 10^−3^
1	0	4		−5.05 × 10^−4^	3	1	0	8.91 × 10^−3^	
1	1	0	−1.05 × 10^−2^		3	1	1	1.66 × 10^−4^	
1	1	1	−1.69 × 10^−2^		3	2	0		−8.40 × 10^−3^
1	1	2	1.32 × 10^−3^		4	0	0	−1.81	−3.01 × 10^−1^
1	1	3	1.47 × 10^−4^		4	0	1	−2.41 × 10^−3^	−1.31 × 10^−2^
1	2	0		6.29 × 10^−2^	5	0	0		3.18 × 10^−1^
1	2	1		6.97 × 10^−4^					
1	2	2		−5.94 × 10^−4^					

Figure 2b shows the potential energy surface *V*(*Q*_*IR*_, *Q*_*LSx*_, *Q*_*LSy*_) represented on (*Q*_*LSx*_, *Q*_*LSy*_) coordinates when the amplitude of *Q*_*IR*_ is zero. The energy contour shows the directional dependence inherited from the *C*_3v_ symmetry. The energy barriers for the AB → AC change along the equivalent *r*_1,_ *r*_2_ and *r*_3_ directions in this potential energy surface are the same as Φ_0_ = 17.3 meV. The difference between Φ_0_ and Φ_NEB_ for the AB → AC stacking change is because the deformation by the in-plane *Q*_*LS*_ mode does not include any out-of-plane relaxation, while the NEB path includes the relaxation from the slight increase (~1.6%) of the interlayer distance, reducing the barrier. It is worth to note that the Φ_0_ rather than the Φ_NEB_ is relevant to the ultrafast switching in the picosecond time scale, while the latter is relevant to the conventional switching in a longer time scale.

The anharmonic coupling effect can be seen from the modulation of the potential energy landscape under the large *Q*_*IR*_ amplitude. Figure 2c,d show the potential energy landscapes when the amplitude of *Q*_*IR*_ was set to ±5.00 Å$\sqrt{\mu }$ along the x- and y-axis, respectively. This amplitude corresponds to the displacement of Mo atoms by ~0.23 Å and that of S atoms by ~0.34 Å in opposite direction along the polarization axis. Due to the deformation, the *C*_3v_ symmetry of the potential surface on the (*Q*_*LSx*_, *Q*_*LSy*_) coordinates was broken, and the energy barriers along the three equivalent directions became different. Under the negative amplitude of *Q*_*IRx*_ = −5.00 Å$\sqrt{\mu }$, the energy barrier along the *r*_1_ direction decreased to 8.6 meV, but that along the other directions increased to 19.4 meV (*r*_2_ direction) and 21.3 meV (*r*_3_ direction), respectively. The energy landscape for the positive amplitude *Q*_*IRx*_ = +5.00 Å$\sqrt{\mu }$ is essentially the same with that for negative amplitude, except for the fact that the energy contour is flipped with respect to the mirror *σ*_1_. For both signs of *Q*_*IRx*_, the coordinate of the potential energy minimum is slightly shifted along the *r*_1_ direction from the origin (*Q*_*LSx*_ = *Q*_*LSy*_ = 0 Å$\sqrt{\mu }$).

By contrast, the amplitude of *Q*_*IRy*_ largely increases the energy barrier along the *r*_1_ direction (31.7 meV at *Q*_*IRy*_ = −5.00 Å$\sqrt{\mu }$, and 23.8 meV at *Q*_*IRy*_ = +5.00 Å$\sqrt{\mu }$). This is accompanied by a slight shift of the potential minimum along the −*r*_1_ (−y) direction. The energy landscape is symmetric with respect to the mirror plane *σ*_1_, and the energy barrier along the *r*_2_ and *r*_3_ directions are reduced (13.4 meV at *Q*_*IRy*_ = −5.00 Å$\sqrt{\mu }$, and 10.1 meV at *Q*_*IRy*_ = +5.00 Å$\sqrt{\mu }$). The change in energy barriers and the shift of the potential minimum indicate that the coupling of *Q*_*LS*_ and *Q*_*IR*_ modes exerts an anharmonic force on the *Q*_*LS*_ mode.

### Dynamics of coupled normal-modes under light pulse

The dynamical behavior of the normal-modes was investigated under light pulse with a specific polarization direction. Since the motion of the *Q*_*IR*_ mode is much faster than that of the *Q*_*LS*_, the *Q*_*LS*_ modes experience the effective potential asserted by the rapidly oscillating *Q*_*IR*_ mode; i.e., time-averaged potential energy surface depending on *Q*_*IR*_(*t*). The dynamics of the nonlinearly coupled modes were simulated by the following coupled equations of motion,

2 $$\begin{array}{rcl}{\ddot{Q}}_{IR} & = & -\,\frac{\partial V({Q}_{IR},{Q}_{LSx},{Q}_{LSy})}{\partial {Q}_{IR}}-{\gamma }_{IR}{\dot{Q}}_{IR}+F(t),\,{\rm{where}}\,{Q}_{IR}=\{{Q}_{IRx},{Q}_{IRy}\},\\ {\ddot{Q}}_{LSx} & = & -\,\frac{\partial V({Q}_{IR},{Q}_{LSx},{Q}_{LSy})}{\partial {Q}_{LSx}}-{\gamma }_{LS}{\dot{Q}}_{LSx},\\ {\ddot{Q}}_{LSy} & = & -\,\frac{\partial V({Q}_{IR},{Q}_{LSx},{Q}_{LSy})}{\partial {Q}_{LSy}}-{\gamma }_{LS}{\dot{Q}}_{LSy},\end{array}$$

where *γ*_*IR*_ and *γ*_*LS*_ are the damping coefficients for each mode, and *F*(*t*) is the optical driving force on the *Q*_*IR*_ mode. We used Gaussian pulse $F(t)={Z}_{IR}^{\ast }{E}_{0}\,\sin \,(\Omega t){e}^{-{t}^{2}/2{\sigma }^{2}}/\sigma \sqrt{2\pi }$, where *E*_0_ is the amplitude of the electric field, σ is the duration of the pulse and Ω is the frequency.

**Figure 3:**
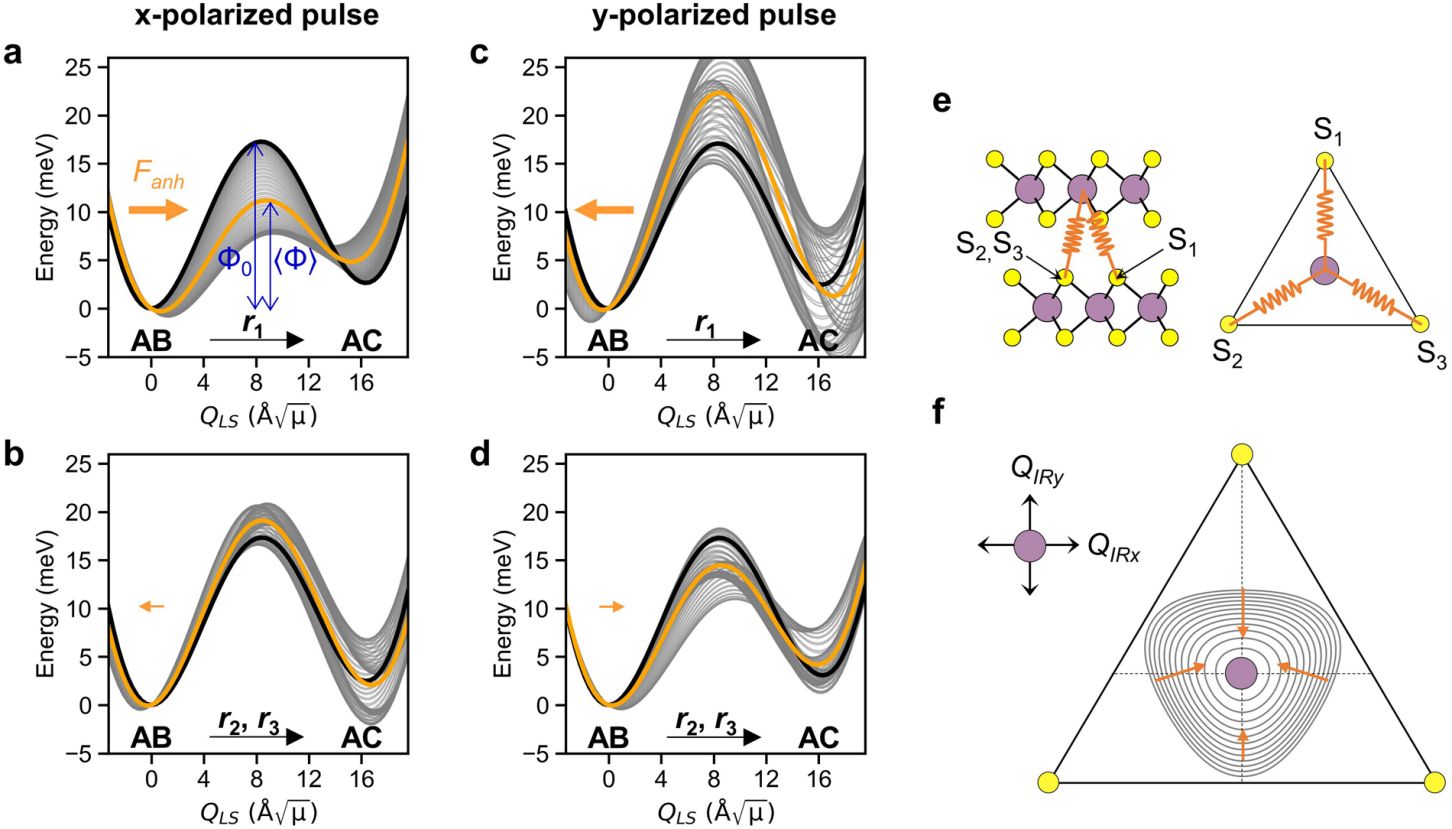
Evolution of potential energy curve on the *Q*_*LS*_ coordinate along the ferroelectric switching directions under (**a**,**b**) x-polarized and (**c**,**d**) y-polarized pulse. Rapidly oscillating *Q*_*IRx*_ under pulse modulates the potential energy curve (grey lines) with respect to the static case at *Q*_*IR*_ = 0 Å$\sqrt{\mu }$ (bold black line). The effective potential energy experienced by the *Q*_*LS*_ is the time-average of the potential energy curves (orange line). 〈Φ〉 is the effective energy barrier under the pulse while Φ_0_ is pristine energy barrier. Direction and relative magnitude of the effective anharmonic force *F*_*anh*_ on *Q*_*LS*_ are depicted by the orange arrow. (**e**) Effective interlayer interaction between the Mo and S sublattices induced by *Q*_*IR*_. (**f**) Contour plot of the interaction energy indicates the direction and relative magnitude of the anharmonic force depending on the polarization direction of *Q*_*IR*_.

Figure 3 shows the evolution of potential energy curve on the *Q*_*LS*_ coordinate along the AB → AC switching directions when the *Q*_*IR*_ mode is resonantly pumped by a pulse with Ω = Ω_*IR*_, *E*_0_ = 34 MVcm^−1^ and σ = 100 fs. Such high intensity of the pulse is required to achieve the large amplitude of *Q*_*IR*_ (~5 Å$\sqrt{\mu }$) in MoS_2,_ in order to explore the strong anharmonic coupling effect. The pulse intensity used in this study is comparable to the that used in the experiment on the high harmonic generation of the single layer MoS_2_^32^. The energy curve (bold black line) corresponds to the static case (*Q*_*IRx*_ = 0 Å$\sqrt{\mu }$), where the energy of the AC stacking is slightly higher than that of the AB stacking because the layer-shearing by the *Q*_*LS*_ mode is not perfectly rigid.

The pulse polarized along the x-axis induces the oscillation of the *Q*_*IRx*_ mode with the amplitude between ± 5.35 Å$\sqrt{\mu }$ by which the potential curve changes (grey line). The time-averaged potential energy (orange line) results in the effective barrier 〈Φ〉 = 11.5 meV along the *r*_1_ direction (Fig. 3a), which is a significant reduction from the 17.3 meV for the static case. The coordinate of the potential minimum was shifted by 0.72 Å$\sqrt{\mu }$ along the *r*_1_ direction, and the energy of the AC stacking slightly increased compared to the static case. Meanwhile, a slight increase of the barrier to 19.2 meV along the *r*_2_ and *r*_3_ directions was observed (Fig. 3b). In contrast, the *Q*_*IRy*_ mode under the y-polarized pulse shows asymmetric vibration between −4.69 Å$\sqrt{\mu }$ and +5.43 Å$\sqrt{\mu }$ in the anharmonic potential due to the lack of the mirror plane perpendicular to the y-axis. This results in an increase of the effective barrier along the *r*_1_ direction to 22.5 meV, and the shift of the potential minimum by 0.36 Å$\sqrt{\mu }$ along the −*r*_1_ direction (Fig. 3c). On the other hand, the effective energy barrier along the *r*_2_ and *r*_3_ directions diminishes to 14.4 meV (Fig. 3d).

The polarization-dependent modulation of the effective potential energy can be explained by the characteristics of the anharmonic coupling terms. The overall trend is captured by the coupling terms in the form of ${Q}_{IR}^{l}{Q}_{LSy}$ with even *l*, which impart an unidirectional anharmonic force on *Q*_*LSy*_ by ${F}_{anh}=-\,\mathop{\sum }\limits_{l}^{even}{c}_{l01}\langle {Q}_{IR}^{l}\rangle$_._ The sign of the coefficient of a quadratic-linear term ${c}_{201}{Q}_{IR}^{2}{Q}_{LSy}$ determines the sign of *F*_*anh*_. The calculated *F*_*anh*_ by the *Q*_*IRx*_ has a positive value of 0.92 meVÅ^−1^*μ*^−1/2^, hence unidirectionally drives the *Q*_*LSy*_ along the +y (*r*_1_) direction (as indicated by the orange arrow in Fig. 3a). The *F*_*anh*_ decreases the effective energy barrier along the *r*_1_ direction by 33%, but increases the energy barrier by 9% along the *r*_2_ and *r*_3_ directions (according to the factor $\cos (2\pi /3){F}_{anh}=-\,(1/2){F}_{anh}$). Compared to *Q*_*IRx*_, the *F*_*anh*_ from *Q*_*IRy*_ is in the opposite direction with a slightly smaller magnitude (−0.87 meVÅ^−1^*μ*^−1/2^). This explains the increase in the energy barrier along the *r*_1_ direction by 30% and the decreases along the *r*_2_ and *r*_3_ directions by 16%.

The polarization-dependent direction of *F*_*anh*_ has a geometrical origin related to the Mo and S sublattices, which are displaced by the *Q*_*IR*_ in the opposite direction (Fig. 1c). The motion of *Q*_*IR*_ modulates the interlayer interaction, which is approximated by the springs connecting the Mo and the S atoms (S_1_, S_2_, S_3_) in the adjacent layers as illustrated in Fig. 3e. The associated interaction energy is $k\sum _{i=1,2,3}\Delta {{d}_{i}}^{2}$, where *k* is spring constant and $\Delta {d}_{i}$ is the change in distances between the Mo and S atoms. The contour plot of the interaction energy in Fig. 3f exhibits an anisotropy arising from the triangular geometry of the atomic arrangement. Particularly, the gradient of contour (orange arrow) indicates the force component along the +y direction when the Mo sublattice oscillates along the x-axis with respect to the S sublattice. On the contrary, the x-component of the force is canceled upon the rapid motion of *Q*_*IRx*_. This simple picture explains the *F*_*anh*_ along the +y direction, and agrees with the selection rule (*l* + *m* = even) for ${Q}_{IRx}^{l}{Q}_{LSx}^{m}{Q}_{LSy}^{n}$ coupling. In contrast, the *Q*_*IRy*_ motion induces net forces along the −y direction due to the imbalance of the force (see the length of orange arrows).

Neither *Q*_*IRx*_ nor *Q*_*IRy*_ imparts such an unidirectional force on *Q*_*LSx*_ since the relevant coupling terms (the ${Q}_{IR}^{l}{Q}_{LSx}$ with even *l*) are absent due to the odd parity of *Q*_*LSx*_. It prohibits the excitation of the interlayer shear along the *r*_2_ and *r*_3_ directions. Although the y-polarized light pulse lowers the energy barrier along the *r*_2_ and *r*_3_ directions, it cannot induce the ferroelectric switching along these directions. Therefore, the most effective way to realize the ferroelectric switching is to use the x-polarized light pulse which induces both the interlayer shear motion and energy barrier lowering along the *r*_1_ direction for the ferroelectric switching to occur.

**Figure 4:**
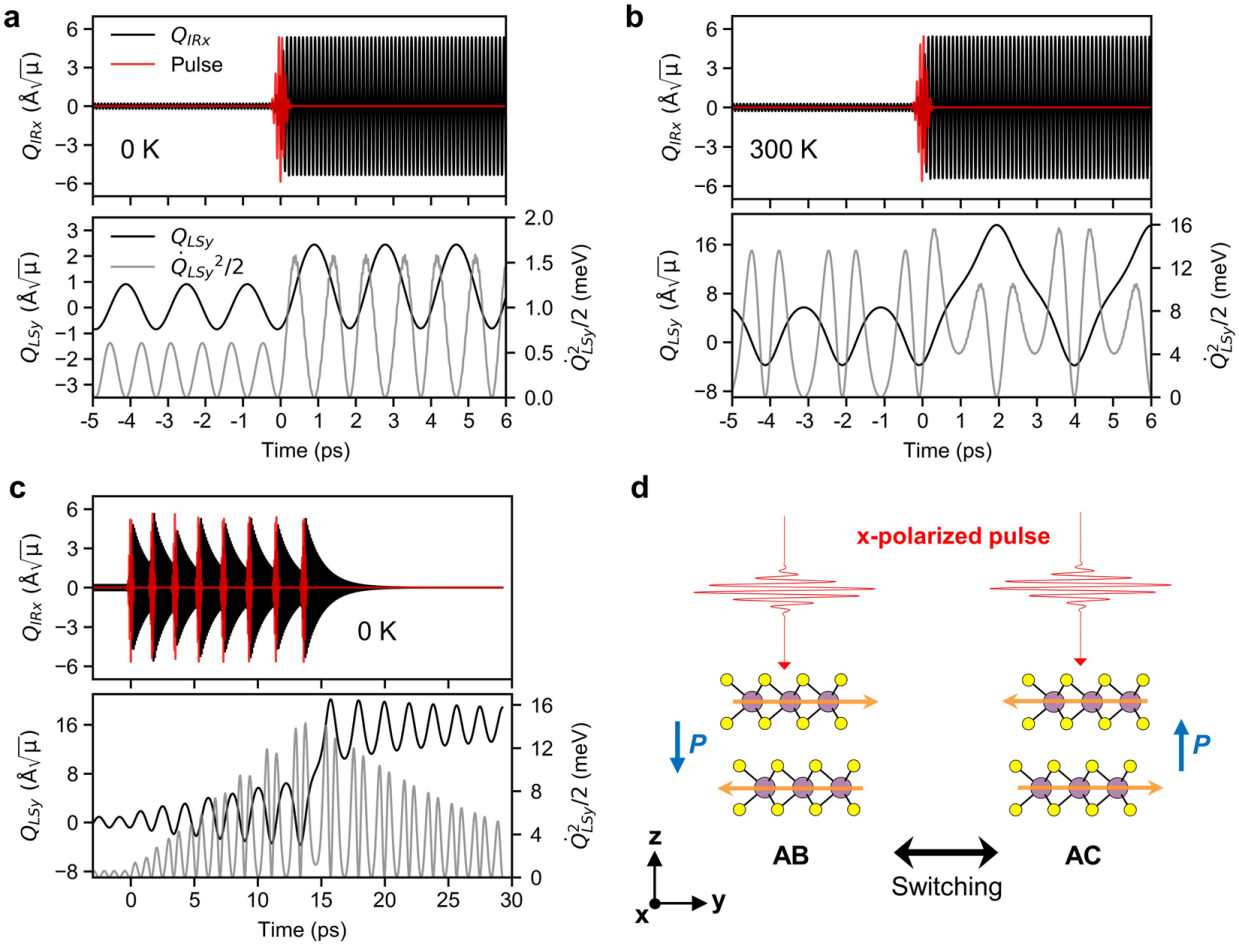
Dynamics of the normal-modes under the x-polarized pulse (**a**,**b**) without and (**c**) with damping. (**a**) Amplification of the *Q*_*LSy*_ mode through the anharmonic force by the pulse at 0 K. (**b**) At 300 K, the *Q*_*LSy*_ has sufficient kinetic energy to overcome the effective energy barrier (〈Φ〉 = 11.5 meV at |*Q*_*IRx*_*|* = 5.35 Å$\sqrt{\mu }$) after the pumping, and oscillates back and forth between AB (*Q*_*LSy*_ = 0 Å$\sqrt{\mu }$) and AC (*Q*_*LSy*_ = 16.29 Å$\sqrt{\mu }$) stackings. (**c**) In the presence of damping, the *Q*_*LSy*_ mode at 0 K overcomes the energy barrier after eight sequential pulses. The AB stacking changes to the AC stacking and does not return due to the dissipation of kinetic energy. (**d**) Schematics of ferroelectric switching through the *Q*_*IRx*_-*Q*_*LSy*_ coupling. The orange arrows indicates the directions of interlayer shear induced by the x-polarized pulse, which are opposite in the AB and AC stackings.

Next, we analyze the dynamics of ferroelectric switching based on the *Q*_*IRx*_-*Q*_*LSy*_ coupling under the x-polarized pulse. First, we considered a case neglecting the damping of normal modes to simply show the essential consequences of the *Q*_*IRx*_-*Q*_*LSy*_ coupling on the dynamics of *Q*_*LSy*_ mode. Figure 4a,b show the motions of *Q*_*IRx*_ and *Q*_*LSy*_ modes at 0 K and 300 K, respectively, without damping under the x-polarized pulse with *E*_0_ = 34 MVcm^−1^ and σ = 100 fs. The initial vibration amplitudes were set as the mean-square-displacement $\sqrt{\langle {Q}_{v}^{2}\rangle }=\sqrt{\frac{\hslash }{{\Omega }_{v}}\frac{2}{{e}^{(\hslash {\Omega }_{v}/{k}_{B}T)}-1}}$ according to the Bose-Einstein distribution at each temperature. The initial vibration of *Q*_*LS*_ was assumed to be aligned to the y-axis by setting the initial coordinate as *Q*_*LSx*_ = 0 Å$\sqrt{\mu }$. This results in the initial amplitudes of *Q*_*IRx*_ = 0.21 Å$\sqrt{\mu }$ and *Q*_*LSy*_ = 0.91 Å$\sqrt{\mu }$ at 0 K, while *Q*_*IRx*_ = 0.28 Å$\sqrt{\mu }$ and *Q*_*LSy*_ = 5.70 Å$\sqrt{\mu }$ at 300 K, respectively.

In Fig. 4a, the *Q*_*IRx*_ and *Q*_*LSy*_ modes oscillate with the harmonic frequencies before the arrival of the pulse at 0 ps. The initial kinetic energy of *Q*_*LSy*_ mode was ${\dot{Q}}_{LSy}^{2}/2$ = 0.6 meV. When *Q*_*IRx*_ was pumped, *Q*_*LSy*_ started to oscillate with larger amplitude with respect to the shifted minimum at *Q*_*LSy*_ = 0.87 Å$\sqrt{\mu }$ (in good agreement with aforementioned 0.72 Å$\sqrt{\mu }$ shift in the effective potential minimum). The pumping does not affect the motion of *Q*_*LSx*_ (the value remains as ~0 Å$\sqrt{\mu }$) as there are no forcing terms on it. The kinetic energy of *Q*_*LSy*_ mode was increased to 1.6 meV by the anharmonic energy flow from the pumped *Q*_*IRx*_ mode. It is noted that the pulse and *Q*_*LSy*_ should be in-phase because the anharmonic force is unidirectional. The vibration of *Q*_*LSy*_ is restricted in a small region because the kinetic energy is still smaller than the effective barrier of 〈Φ〉 = 11.5 meV under the oscillating *Q*_*IRx*_. In contrast, the oscillatory curve of *Q*_*LSy*_ mode at 300 K in Fig. 4b shows slight modulations in shape and frequency by the onset of anharmonicity of *Q*_*LSy*_. The kinetic energy of *Q*_*LSy*_ mode was 13.4 meV which is yet below the static energy barrier Φ_0_ = 17.3 meV, but higher than the effective barrier 〈Φ〉 = 11.5 meV under the pulse. When *Q*_*IRx*_ mode was pumped, *Q*_*LSy*_ mode jumped over the barrier and oscillated with colossal amplitude between −3.79 Å$\sqrt{\mu }$ and +19.19 Å$\sqrt{\mu }$. The vibration corresponds to the repetitive interconversion between AB and AC stackings, due to the absence of damping.

Secondly, a more realistic model that includes the damping of the normal modes was considered. The damping coefficients of *γ*_*IR*_ and *γ*_*LS*_ were taken as 2% of the harmonic frequencies, which are similar to the experimental values^21^. In Fig. 4c, the *Q*_*IRx*_ and *Q*_*IRy*_ modes initially oscillate with small amplitudes at 0 K until the arrival of the first pulse at 0 ps (damping is turned on at 0 ps). The eight sequencial pulses are applied to substantially amplify the *Q*_*LS*y_ mode from the zero-point vibration at 0 K. The time interval between the subsequent pulses is gradually increased by ~4% from the 1/Ω_*LS*_ ~ 1.6 ps for the phase matching between the pulse and *Q*_*LSy*_ mode, considering the increase in the period of *Q*_*LS*_ mode due to anharmonicity. Upon each cycle of pulse irradiation, the *Q*_*LSy*_ mode is coherently amplified by gaining kinetic energy. After the eight pulses are irradiated, the *Q*_*LSy*_ mode has sufficient kinetic energy and jump over the effective barrier which is reduced by the *Q*_*IRx*_ mode. Once the initial AB stacking sequence changes to the AC stacking, it maintains the AC stacking due to the dissipation of the kinetic energies of the vibrations. This corresponds to the AB → AC ferroelectric switching. The opposite switching operation, AC → AB, can be performed by the same optical input as illustrated in Fig. 4d. The direction of *F*_*anh*_ on *Q*_*LSy*_ mode in the AC stacking is reversed (−*r*_1_ direction) with respect to that (*r*_1_ direction) in the AB stacking. The optical parameters of pulses (e.g. *E*_0_ and σ) used in this study might be optimized further for more efficient switching, for instance, via pulse shaping techniques^37^.

## Conclusion

In summary, the polarization switching mechanism of the bilayer 3R MoS_2_ whose direction of the polarization is reversed by the change of the stacking sequence was investigated. The ferroelectric switching was achieved by driving the interlayer shear mode through the anharmonic energy flow from the optically pumped infrared mode. Remarkably, due to the selection rule from the crystal symmetry of MoS_2_, the degenerate interlayer shear mode can only be driven along its armchair axis whether the infrared mode is pumped along the zigzag or armchair axis. However, the optical pulse should be polarized along the zigzag axis for successful switching since the direction of anharmonic force is aligned with the switching direction. The coherent light pulses can amplify the interlayer shear mode substantially and unidirectionally, displacing the stacking sequence into the opposite polarization. The scheme for optical modulation of the stacking structure can be applied to other 2D materials exhibiting the interlayer shear mode to explore various stacking-dependent properties in a dynamical manner.

## Methods

The density functional theory calculations were performed using Vienna Ab-initio Simulation Package (VASP)^38,39^. The projector-augmented wave (PAW) method^40^ and a cut-off energy of 500 eV were used with the valence electron configurations of Mo[4s^2^4p^6^5s^2^4d4] and S[3s^2^3p4], respectively. The generalized gradient approximation^41^ with Grimme’s D3 scheme^42^ was used to describe the van der Waals interaction. The bilayer structure was simulated by the supercell containing ~40 Å of vacuum layer to avoid the artificial interaction between periodic images. The structures were fully relaxed using 24 × 24 × 1 k-mesh until the residual forces on the atoms were less than 0.001 eVÅ^−1^. Spontaneous polarization was calculated using Berry phase method^43^. The phonon calculation was performed using PHONOPY code^44^ using 3 × 3 × 1 supercell and 8 × 8 × 1 k-mesh.
